# Comparative effectiveness of image-guided radiotherapy for non-operated localized esophageal squamous cell carcinoma patients receiving concurrent chemoradiotherapy: A population-based propensity score matched analysis

**DOI:** 10.18632/oncotarget.12250

**Published:** 2016-09-26

**Authors:** Chia-Chin Li, Chih-Yi Chen, Chun-Ru Chien

**Affiliations:** ^1^ Cancer Center, Department of Radiation Oncology, China Medical University Hospital, School of Medicine, College of Medicine, China Medical University, Taichung, Taiwan; ^2^ Division of Thoracic Surgery, Department of Surgery, Chung Shan Medical University, Chung Shan Medical University Hospital, Taichung, Taiwan

**Keywords:** concurrent chemoradiotherapy, esophageal squamous cell carcinoma, image-guided radiotherapy

## Abstract

**Background:**

Although concurrent chemoradiotherapy (CCRT) coupled with image-guided radiotherapy (IGRT) is associated with a theoretical benefit in non-operated localized esophageal squamous cell carcinoma (NOL-ESCC) patients, there is currently no clinical evidence to support this.

**Results:**

The study population in the primary analysis comprised 866 patients who were well balanced in terms of their co-variables. The HR for mortality when group A was compared with group B was 0.82 (95% confidence interval, 0.7–0.95). SA revealed that the result was moderately sensitive.

**Materials and Methods:**

Eligible patients diagnosed between 2008 and 2013 were identified in the Taiwan Cancer Registry. A propensity score-matched cohort was constructed [1:1 in groups A (with IGRT) and B (without IGRT)] to balance any observable potential confounders. The hazard ratio (HR) for mortality was compared between groups A and B during the follow-up period. Sensitivity analyses (SA) were performed to evaluate the robustness of the findings regarding the selection of confounders and a potential unobserved confounder.

**Conclusions:**

The current results provide the first clinical evidence that CCRT coupled with IGRT is associated with better overall survival when compared with CCRT without IGRT in NOL-ESCC patients. However, this study should be interpreted with caution given its non-randomized nature and the moderate sensitivity of the data. Further studies are needed to clarify this finding.

## INTRODUCTION

Esophageal cancer is a major cause of cancer-related mortality worldwide [1]. Squamous cell carcinoma (SqCC) is the most prevalent form, particularly in non-Western countries [1, 2]. Definitive concurrent chemoradiotherapy (CCRT) is one of the standards of care for localized esophageal SqCC cases beyond the early stage [3]. Randomized trials have compared definitive chemoradiotherapy with surgery with or without chemoradiotherapy and reported comparable survival [4–6]. Image-guided radiotherapy (IGRT) is an advanced radiotherapy technology with great potential to improve patient outcomes via improved radiotherapy delivery [7]. However, little high-level evidence is available to support a clinical benefit for IGRT [7, 8].

Some reviews have suggested that IGRT is likely to improve the prognosis of patients with esophageal cancer [9, 10]; however, no clinical evidence for this has been provided. We searched PubMed using the terms “(esophageal cancer) AND [(radiation therapy) OR (cancer radiotherapy)] AND (image-guided radiotherapy) AND (survival)” on April 17, 2016, but did not find relevant clinical evidence except one report stating that IGRT was feasible in a Phase 2 study [11]. Since the clinical benefit of IGRT in esophageal cancer is unclear, the current study investigated the survival outcomes of non-operated localized esophageal SqCC (NOL-ESCC) patients receiving CCRT either with or without IGRT using a population-based propensity-score (PS) matched analysis.

## RESULTS

### Study population

**Figure 1:**
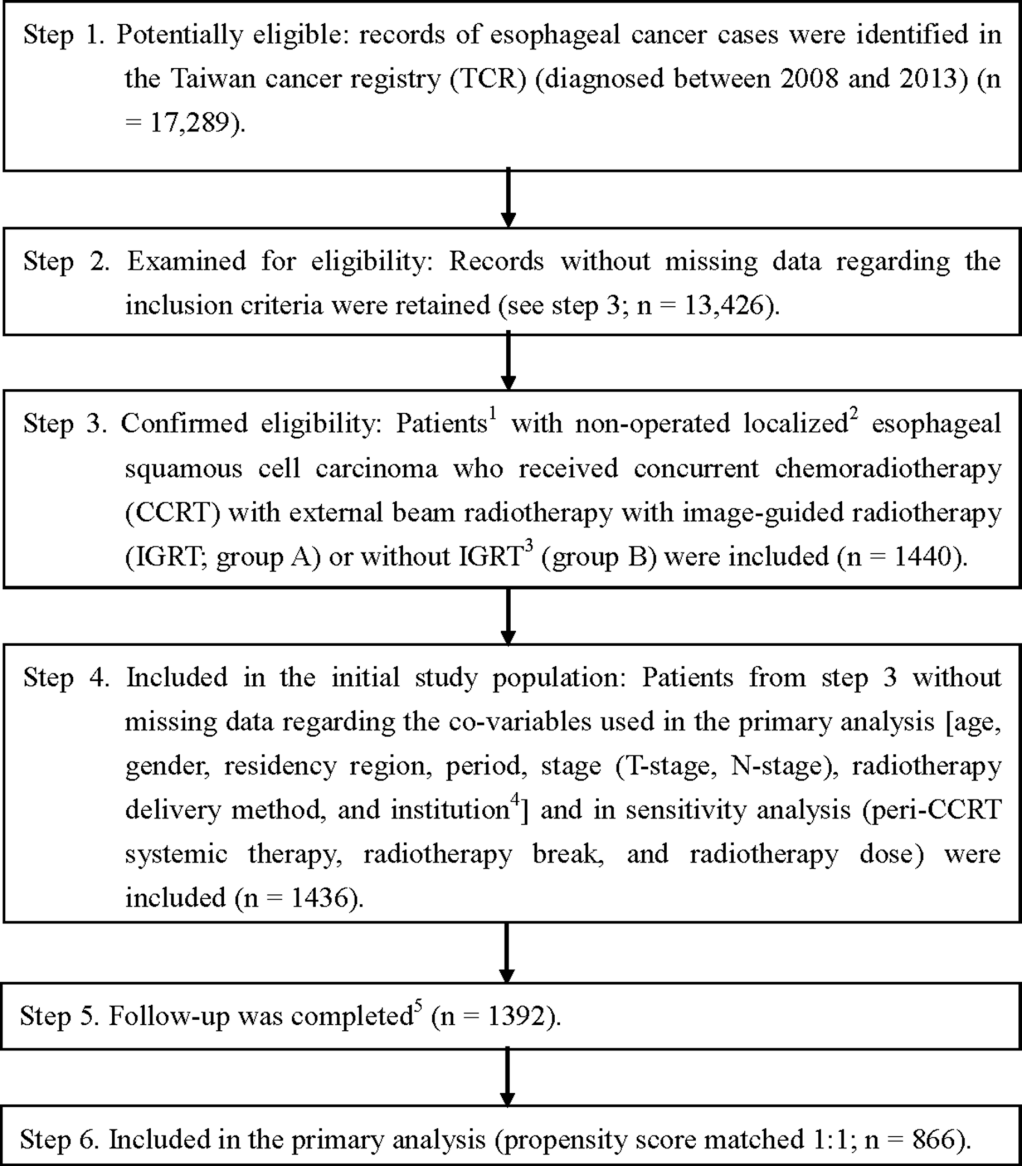
Study flow chart 1 Only patients treated (class 1–2) by any single institution were included to ensure data consistency. 2 Sixth American Joint Committee on Cancer staging clinical stage 2–4a (2008–2009) or Seventh staging stage 2–3 (2010–2013). 3 Patients who were either treated at an institution without IGRT use during the study period or whose radiotherapy started before the first IGRT case at each institution were excluded. 4 Classified as high volume (at least 69 eligible patients during the study period) or low volume. 5 Without missing information in the TCR and death registry.

As shown in Figure 1, 1,440 eligible cancer patients who received CCRT with external beam radiotherapy between 2008 and 2013 were identified from the 17,289 esophageal cancer records in the Taiwan Cancer Registry (TCR). They were divided into two groups: those who received CCRT with IGRT (group A) and those who received CCRT without IGRT (group B). The final PS-matched study population used in the primary analysis included 866 patients. The patient characteristics are described in Table 1. The groups were well balanced regarding co-variables; small standardized differences (< 0.25) were seen for all co-variables [12].

**Table 1:** Characteristics of the matched study population in the primary analysis

		Group A: with IGRT	Group B: without IGRT	
		number (%)^#^ or mean (SD)^#^	number (%)^#^ or mean (SD)^#^	Standardized difference (rounded)
Age	< 65	334 (77.14)	331 (76.44)	0.016
	≥ 65	99 (22.86)	102 (23.56)	
Gender	female	17 (3.93)	16 (3.7)	0.012
	male	416 (96.07)	417 (96.3)	
Residency	non-north	295 (68.13)	290 (66.97)	0.025
	north	138 (31.87)	143 (33.03)	
T-stage	T1–T2	71 (16.4)	71 (16.4)	0
	T3–T4	362 (83.6)	362 (83.6)	
N-stage	positive	392 (90.53)	393 (90.76)	0.008
	negative	41 (9.47)	40 (9.24)	
RT delivery	3DCRT	7 (1.62)	6 (1.39)	0.019
	IMRT	426 (98.38)	427 (98.61)	
Institution	high-volume	223 (51.5)	226 (52.19)	0.014
	low-volume	210 (48.5)	207 (47.81)	
Period	2008–2009	40 (9.24)	40 (9.24)	0
	2010–2013	393 (90.76)	393 (90.76)	

Abbreviations:

# rounded to the second decimal place;

IGRT, image-guided RT; 3DCRT, 3D conformal RT; IMRT, intensity-modulated RT; SD, standard deviation.

### Primary analysis

**Figure 2:**
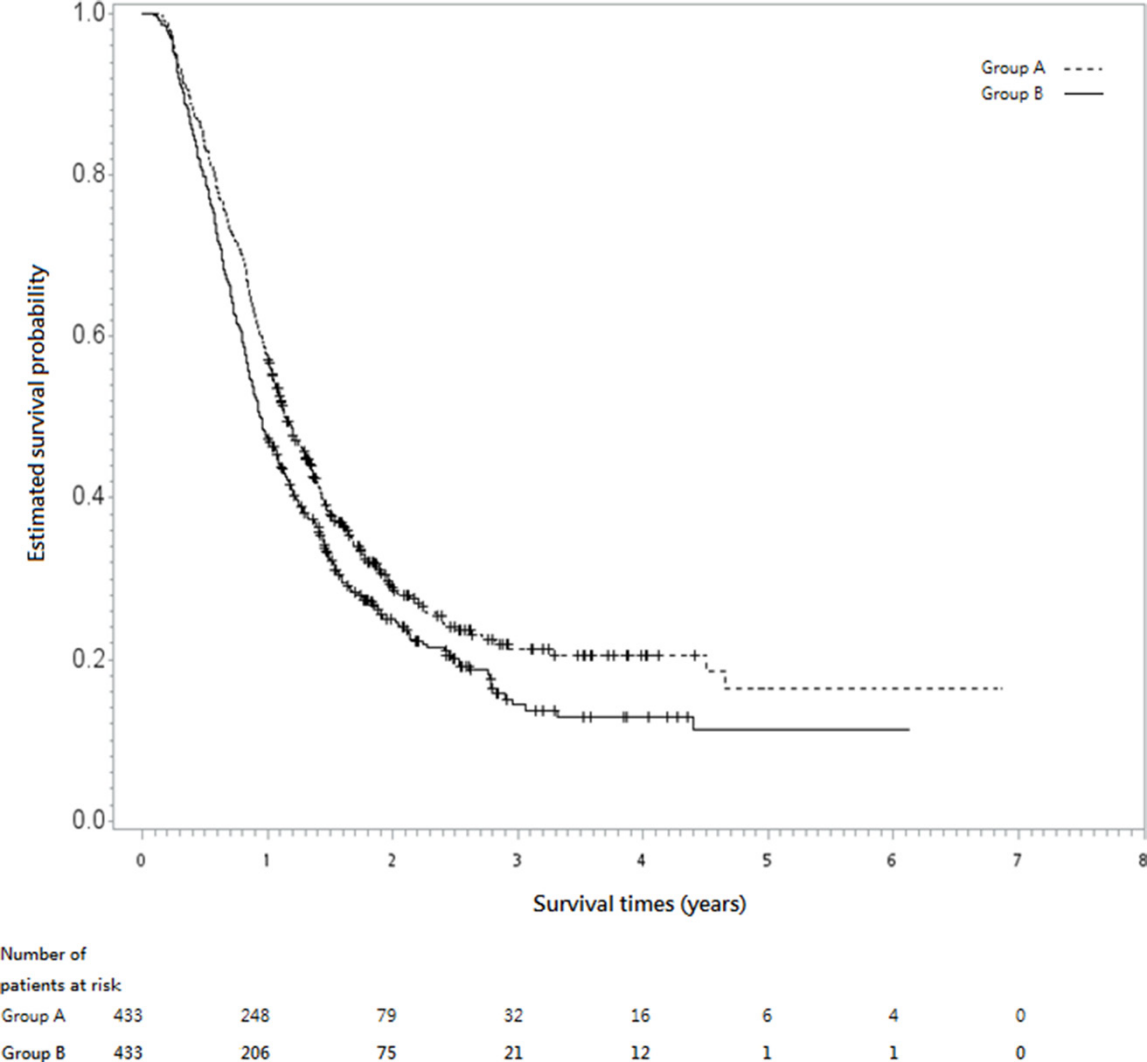
Kaplan-Meier survival curve (in years) The dotted line represents group A (with image-guided radiotherapy, IGRT) and the solid line shows group B (without IGRT).

After a median follow-up period of 12.65 months (range, 1.1–83.5), the hazard ratio (HR) for death when group A (IGRT) was compared with group B (without IGRT) was 0.82 [95% confidence interval (CI), 0.7–0.95, *p* = 0.0072]. The 5-year overall survival rates were 16% in group A vs. 11% in group B. Figure 2 shows the Kaplan-Meier survival curve for overall survival (OS).

### Sensitivity analysis (SA)

In sensitivity analysis 1 (SA-1), well-balanced co-variables were seen after PS-matching when additional “variables of ambiguous status” (see Methods section for a description) were also included (Table 2). The HR for death when group A was compared with group B was 0.95 (95% CI, 0.82–1.1; *p* = 0.5). In sensitivity analysis 2 (SA-2) regarding the potential impact of an unmeasured confounder on the findings in our primary analysis, the data revealed that, if there was an unmeasured binary confounder that increased the odds of IGRT (vs. without IGRT) to 5.5% instead of zero, the conclusion that IGRT was more effective remained statistically significant (Table 3; *p* < 0.05). However, if an unmeasured binary confounder increased the odds of IGRT to at least 6%, the observed effectiveness of IGRT would no longer be statistically significant (*p* > 0.05).

**Table 2:** Characteristics of the matched study population in SA-1

		Group A: with IGRT		Group B: without IGRT		
		number or mean (SD)^#^	(%)^#^	number or mean (SD)^#^	(%)^#^	Standardized difference (rounded)
Age	< 65	336	(74.5)	347	(76.94)	0.057
	≥65	115	(25.5)	104	(23.06)	
Gender	female	22	(4.88)	25	(5.54)	0.03
	male	429	(95.12)	426	(94.46)	
Residency	non-north	306	(67.85)	301	(66.74)	0.024
	north	145	(32.15)	150	(33.26)	
T-stage	T1–T2	81	(17.96)	80	(17.74)	0.006
	T3–T4	370	(82.04)	371	(82.26)	
N-stage	positive	407	(90.24)	406	(90.02)	0.007
	negative	44	(9.76)	45	(9.98)	
RT delivery	3DCRT	9	(2)	6	(1.33)	0.052
	IMRT	442	(98)	445	(98.67)	
Institution	high-volume	230	(51)	233	(51.66)	0.013
	low-volume	221	(49)	218	(48.34)	
Period	2008–2009	39	(8.65)	39	(8.65)	0
	2010–2013	412	(91.35)	412	(91.35)	
Peri-CCRT systemic therapy	with	123	(27.27)	123	(27.27)	0
	without	328	(72.73)	328	(72.73)	
RT break	≤ 1 week	310	(68.74)	311	(68.96)	0.005
	> 1 week	141	(31.26)	140	(31.04)	
RT dose (Gy)		56	(10.78)	56.16	(10.4)	0.015

Abbreviations:

# rounded to the second decimal place;

3DCRT, 3D conformal RT; CCRT, concurrent chemoradiotherapy; IGRT, image-guided RT; IMRT, intensity-modulated RT; RT, radiotherapy; SD, standard deviation.

**Table 3:** Sensitivity analysis

Increased odds of IGRT (vs. without IGRT) by unmeasured confounders	*p*-value
2%	0.023
4%	0.036
5%	0.044
5.5%	0.049
6%	0.054
8%	0.077

## DISCUSSION

This population-based PS-matched analysis provides the first clinical evidence that CCRT coupled with IGRT is associated with better OS than CCRT without IGRT in NOL-ESCC patients.

IGRT improves awareness of set-up error and internal motion to ensure that radiation hits the target rather than normal tissue [13]. Therefore, the current results are consistent with this theoretical benefit of IGRT, suggesting improved outcomes as a result of enhancing the accuracy of delivery of radiotherapy [7, 8]. This is consistent with the results reported for other disease sites [14–18].

However, this study should not be interpreted as conclusive given its non-randomized design and the moderate sensitivity of the SA. In SA-1, which included “variables of ambiguous status”, IGRT was still favorable (HR < 1). In SA-2, which included “the assumption of no unobserved confounder”, a small imbalance in potential unmeasured confounder(s) amounting to a 6% increase could have caused the results to be no longer statistically significant. Therefore, a randomized controlled study is warranted to confirm these results, although no relevant trials were identified when www.clinicaltrials.gov was searched using the term “image-guided radiotherapy esophageal cancer” on April 17, 2016.

There are several limitations to the current study. First, the potential for an unobserved confounder was a limitation of the non-randomized study design, as mentioned above. There is also particular concern regarding the selection of IGRT, which was non-randomized. IGRT is not reimbursed by the National Health Insurance in Taiwan. To our knowledge, the selection of IGRT was at the discretion of the in-charge radiation oncologist and was provided free without additional charges in some hospitals; however, at other hospitals it was more often provided after out-of-pocket payments by patients. However, data regarding the socioeconomic status of the patients were not available in the TCR, although this might be partly related to patient residency. Other potential confounders, such as performance status, comorbidities, and chemotherapy dose/regimen, were not included in the adjustment because they were not available in the TCR. Therefore, a sensitivity analysis (SA-2) was performed regarding potential unmeasured confounders, as suggested previously [19]. Second, the impact of novel modalities such as carbon ions [20] or new targeted therapies [21] was not considered in this study. Finally, the intervention (IGRT) might have been heterogeneous. To our knowledge, several different forms of IGRT are available in Taiwan, including but not limited to cone beam computed tomography, kV imaging, and mV imaging, which could not be differentiated in the TCR. However, it is unclear whether these various technologies lead to different clinical benefits [13]. Therefore, the current study reflects the current population-level effectiveness of IGRT in Taiwan, but the data might not be valid for other countries or regions.

In conclusion, this study provides the first clinical evidence that CCRT coupled with IGRT is associated with better OS than CCRT without IGRT in NOL-ESCC patients. The results should be interpreted with caution given the non-randomized design and the moderate sensitivity of the SA. Further studies are needed to clarify this finding.

## MATERIALS AND METHODS

### Data source

The data were obtained from the TCR and a registry of deaths. The TCR was established in 1979. The central cancer registry was reformed in 2002 to include details regarding the stage at diagnosis and the first course of treatment (termed the long-form database). Esophageal cancer has been included in this mandatory nationwide “long-form database” since 2008. All recordings are made by the professional cancer registrar(s) in each hospital in collaboration with relevant physicians, and are further reviewed by the TCR. The excellent quality (97% completeness) of the TCR data has been confirmed [22].

### Study population and design

The study flow chart is depicted in Figure 1, as suggested in the STROBE statement [23]. The study population consisted of non-operated localized esophageal SqCC patients who were diagnosed between 2008 and 2013 and received CCRT with external beam radiotherapy. The date of diagnosis in the cancer registry was adopted as the index date and the explanatory variable of interest was treatment with or without IGRT (groups A and B, respectively). Documentation regarding the use of IGRT was based on data in the TCR. The different forms of IGRT (such as cone beam computed tomography, kV imaging, or mV imaging) [13] could not be differentiated based on the data in the TCR. Patients who were treated at an institution without IGRT use during the study period or whose radiotherapy started before the first IGRT case was treated at each institution were excluded to ensure that groups A and B could be compared accurately. Data regarding co-variables were also collected to adjust for potential non-randomized treatment selections (see below). The survival status of each patient was obtained from the death registry (from follow-up until December 31, 2014). Then, a PS-matched sample was constructed based on the estimated PS with the above co-variables, and survival analysis was performed to evaluate the effects of CCRT with and without IGRT. The on-period was matched exactly, both because staging changed in 2010 and to reduce potential lead-time bias. To overcome the inherent limitations of retrospective studies, a PS approach was adopted instead of the traditional regression methods, as advocated in the literature [24]. Another advocated approach (instrumental variable) was not adopted because a valid instrument could not be identified with confidence in the TCR [25].

### Other explanatory co-variables

This study included patient demographics (age, gender, residency region) and disease/treatment parameters (treatment period, clinical T- and N-stage, radiotherapy delivery method, and institution) as co-variables that were adjusted in the primary analysis. Three “variables of ambiguous status”, which were “perhaps slightly affected by the treatment, but plausibly standing in as a surrogate for an important co-variable that was not measured,” were included in the sensitivity analyses [26]: the use of peri-CCRT (i.e., induction or consolidative) systemic therapy, radiotherapy break, and radiotherapy dose (see “Statistical and sensitivity analyses”). The selection and definition of these factors were based on our experience in clinical care and previous studies using data from the TCR [27–31]. The co-variables were defined as follows: age was classified as ≥ 65 years or not, patient residency region was classified as northern Taiwan or elsewhere, T-stage was classified as T1–T2 or T3–T4, N-stage was classified as positive [N1M0 or N0-1M1a (2008–2009); N1-N3 (2010–2013)] or negative, the period was classified as 2008–2009 or 2010–2013, external beam radiotherapy delivery was classified as 3D conformal radiotherapy (3DCRT) or intensity-modulated radiotherapy (IMRT), institutions were classified as high-volume institutions vs. low volume institutions using a cut-off point that roughly split the study sample equally, peri-CCRT systemic therapy was classified as yes or no, and the interval of radiotherapy break was classified as > 1 week or ≤ 1 week.

### Effectiveness assessment

The survival status at the end of the follow-up period was obtained from the registry of deaths. This information was used to compare the OS of patients in groups A and B.

### Statistical and sensitivity analyses

Statistical analyses were performed using SAS 9.3 (SAS Institute, Cary, NC, USA), and STATA 12.1 (StataCorp LP, College Station, TX, USA) was used for matching. Tabulation and standardized differences were used to assess the balance of the co-variables between the PS-matched groups. The HR of mortality between groups A and B during the entire follow-up period was compared using a robust variance estimator [19]. In sensitivity analysis 1 (SA-1), a PS sample match was constructed using the co-variables used in the primary analysis plus the three mentioned above in “variables of ambiguous status”. In sensitivity analysis 2 (SA-2), the robustness of the findings of the primary analysis was examined to identify any potential unmeasured confounder(s). Under the assumption of “no unmeasured confounder”, the probability of receiving either treatment should be the same after PS matching. However, if there was an unmeasured confounder that was associated with both treatment selection and outcome, then the true probability of receiving treatment might differ for a factor (labeled Γ) even after PS matching. Therefore, sensitivity analysis was used as suggested previously [19] to assess the statistical significance of the treatment effect that would be observed had this unmeasured confounder been accounted for at various levels of Γ. Therefore, the robustness of the results could be tested at various levels of violation of the “no unmeasured confounder” assumption. This study was approved by the Research Ethics Committee, National Health Research Institutes [EC1041006-E].
